# Alitretinoin reduces erythema in inherited ichthyosis

**DOI:** 10.1186/s13023-018-0783-9

**Published:** 2018-04-04

**Authors:** Giuliana Onnis, Christine Chiaverini, Geoffroy Hickman, Isabelle Dreyfus, Judith Fischer, Emmanuelle Bourrat, Juliette Mazereeuw-Hautier

**Affiliations:** 1 Reference Centre for Rare Skin Diseases, Dermatology Department, CHU Larrey, Paul Sabatier University, 24, Chemin de Pouvourville, 31400 Toulouse, Cedex 09 France; 2 Department of Dermatology, L’Archet Hospital, CHU de Nice, Nice, France; 3 Department of Dermatology, Reference center for rare skin diseases MAGEC, Saint Louis Hospital, Paris, France; 4 Institute of Human Genetics, University Medical Center Freibur, Freiburg, Germany

**Keywords:** Inherited ichthyosis, Alitretinoin, Retinoids, Therapy, Efficacy, Tolerance, Side-effects

## Abstract

**Background:**

Acitretin is the main retinoid used to treat severe inherited ichthyosis. Alternatives may be considered if it results ineffective or there are side-effects, or for women of childbearing age.

Our objective is evaluation of the effects and tolerance of alitretinoin.

An observational retrospective multicentric study was designed to analyse patients with inherited ichthyosis treated by alitretinoin.

**Results:**

A total of 13 patients were included, 11 of whom were receiving acitretin at inclusion. The main reason for switching to alitretinoin was a desire for pregnancy, but also because of side-effects or unsatisfactory efficacy. Starting dose was 10 mg/day, increased to 20 or 30 mg/day. Alitretinoin seemed to be more effective than acitretin at reducing erythema, but was less effective at reducing scaling or hyperkeratosis. Global efficacy was considered low for two patients, moderate for nine, and high for two. Treatment was well-tolerated, except for one patient who presented with benign intracranial hypertension leading to discontinuation of treatment.

**Conclusions:**

Alitretinoin may be suitable for hereditary ichthyosis with prominent erythema, especially for women of childbearing age.

## Background

Inherited ichthyosis (II) is a heterogeneous group of genetic diseases characterized by scaling that affects the entire skin, and is often associated with hyperkeratosis and skin inflammation [1]. Other symptoms include heat intolerance, pruritus, pain, conductive deafness, and ocular complications, e.g., caused by ectropion. II is primarily a monogenic disease, with more than 40 gene mutations identified (to date) that cause a defective skin barrier [2]. Its classification is based on its clinical presentation and is basically distinguished between non-syndromic (including common ichthyoses, autosomal recessive congenital ichthyosis, keratinopathic ichthyosis, and other forms) and syndromic ichthyoses [2]. II usually has a strong impact on quality of life and requires lifelong treatment [3, 4].

As yet there is no curative therapy. Topical agents represent the first-line treatment. If they are not fully effective or if skin-care is very burdensome, oral retinoids may be considered. There are three types of oral retinoids available: acitretin, alitretinoin and isotretinoin. Etretinate is no longer available in most countries.

Acitretin is the main retinoid prescribed and the only one approved in Europe to treat II [5]. The risk/benefit balance of acitretin is considered favorable, but teratogenesis in women wishing to become pregnant is the main concern, with contraindications to pregnancy for 3 years after acitretin-treatment is discontinued. Acitretin may also not be fully efficacious and can be associated with other adverse effects or, rarely, hypersensitivity [6].

Alitretinoin (Toctino®) (9 cis-retinoic acid) is a recent oral retinoid that has more rapid clearance and, consequently, a shorter teratogenic risk (only 1 month after drug discontinuation). It is approved in Europe for severe chronic hand eczema that is unresponsive to potent topical corticosteroids [7, 8]. In this setting, the recommended dosage is 10 or 30 mg once daily, with dose adjustments based on efficacy and tolerability. There are only a few literature reports in II patients [9–11]. We report here on the effect and tolerability to alitretinoin in a series of patients with II.

## Methods

This observational retrospective multicentric study was performed in three French centers that specialized in II (Toulouse, Paris, Nice). All patients with II and treated with alitretinoin (ongoing or previous treatment) between January 2014 and June 2017 were included. This study was approved by the statutory and ethics authorities according to new French regulations (Loi Jardé). Data were obtained from medical records and photographs.

The clinical parameters (erythema, scaling or hyperkeratosis, palmoplantar keratoderma, ectropion) were assessed using visual analog scales (VAS: 0-10) at two different time-points: before and after introducing alitretinoin (patients were assessed after at least 1 month receiving the maximum dosage). Prominent clinical sign was identified for each patient based on the physician’s opinion and the patient’s opinions/ impact on quality of life. The efficacy of alitretinoin was defined as high, moderate, or low according to if a decrease in the prominent clinical sign was ≥ 3, between 2 and 1, or < 1, respectively. The patient was also asked to evaluate the global efficacy of alitretinoin (high, moderate, or low/absent), and any side-effects were recorded.

## Results

A total of 13 patients were included in this study; their characteristics are described in Table 1.

**Table 1:** Characteristics of the 13 cases inherited ichthyosis treated by alitretinoin

Patients	Gender	Age at start of study	Mutated gene	Form of II	Acitretin treatment before starting AL	Reasons for introducing AL	Maximum dosage of AL (mg/day)	AL duration (months)	Side effects of AL	Treatment change for due to side effects	Causes of AL withdrawal	Monitoring
1	M	24	*ICTHYN*	LI	Yes	AC IE	30	36	Moderate headache	RD	SE IE at 10 mg/day	Lipid, liver
2	F	33	*CYP4F22*	LI	Yes	Desire for pregnancy	10	1	–	–	–	Lipid, liver, *β*-HCG
3	F	32	*ICTHYN*	LI	Yes	Desire for pregnancy	30	12	Moderate headache	RD	SE IE at 10 mg/day	Lipid, liver, *β*-HCG
4	F	34	*ICTHYN*	LI	Yes	Desire for pregnancy	30	36	–	–	–	Lipid, liver, *β*-HCG
5	F	36	*ICTHYN*	LI	Yes	Desire for pregnancy	30	24	–	–	–	Lipid, liver, *β*-HCG
6	F	28	*SPINK5*	NS	No	Desire for pregnancy	30	6	Benign intracranial hypertension	S	SE	Lipid, liver, *β*-HCG
7	M	12	*ABCA12*	CIE	Yes	AC SE (mucous dryness)	10	18	–	–	–	Lipid, liver, *β*-HCG
8	M	38	Not identified	CIE	Yes	AC SE (myalgia)	30	12	–	–	–	Lipid, liver, *β*-HCG
9	F	35	*ABCA12*	CIE	No	Desire for pregnancy	30	24	Moderate headache	–	–	Lipid, liver, *β*-HCG
10	F	30	*POMP*	KLICK	Yes	Desire for pregnancy	10	16	–	–	–	Lipid, liver, *β*-HCG,TSH,T4 X-rays
11	F	25	*TGM1*	LI	Yes	Desire for pregnancy AC IE	30	15	Myalgia	–	–	Lipid, liver, *β*-HCG,TSH,T4 X-rays
12	F	23	ongoing	LI	Yes	Desire for pregnancy AC IE	20	2	Severe headache Mucous oral dryness	–	–	Lipid, liver, *β*-HCG,TSH,T4,X-rays
13	F	28	*GJB3*	EK	Yes	Desire for pregnancy	30	2	Mucous oral dryness	–	–	Lipid, liver, *β*-HCG,TSH

*AL* alitretinoin, *AC* acitretin, *CIE* congenital ichthyosiform erythroderma, *EK* erythrokeratoderma, *IE* insufficient efficacy, *KLICK* keratosis linearis ichthyosis congenital keratoderma, *LI* lamellar ichthyosis, *M* months, *NS* Netherton syndrome, *RD* reduced dosage, *S* stopped treatment, *SE* side-effects, *Y* years

There were three males and ten females (all of childbearing age); overall median age was 30 years [range: 12-38]. None had a significant previous medical history. Ten patients suffered from congenital ichthyosis, seven of them having lamellar ichthyosis (*Ichthyn: n* 4, *CYP4F22:* n 1, *TGM1: n* 1, ongoing analysis: n 1) and three cases of congenital ichthyosiform erythroderma (two patients who carried *ABCA12* mutations, and one who had no identified mutation). The remaining three patients had Netherton syndrome (one case due to *SPINK5* mutations), erythrokeratoderma (one case due to *GJB3* mutation), and KLICK (keratosis linearis with ichthyosis congenita and sclerosing keratoderma) syndrome (one case carrying *POMP* mutation). The prominent sign was scaling in five patients (five with lamellar ichthyosis with a VAS between 5 and 9) and erythema for the other eight patients (two with lamellar ichthyosis, one case of Netherton syndrome, three with congenital ichthyosiform erythroderma, one with KLICK syndrome, one with erythrokeratoderma; VAS was between 5 and 8) (Table 2).

**Table 2:** Effects of alitretinoin on 13 patients with inherited ichthyosis

Patients	Form of II	Prominent sign	Alitretinoin’ efficacy on the prominent sign	Dosage of AL at the time of the final VAS evaluation (mg/day)	Global efficacy	VAS Scale (0-10) Before - during AL	VAS erythema (0-10) Before - during AL	VAS Palmoplantar keratoderma (0-10) Before - During AL	VAS Ectropion (0-10) Before - During AL
1	LI	S	Low	10	Low	5-7	0-0	4-4	0-0
2	LI	E	High	10	High	5-3	5-1	2-2	0-0
3	LI	S	Low	10	Low	5-7	0-0	4-4	0-0
4	LI	S	Moderate	30	Moderate	5-3	0-0	3-2	0-0
5	LI	E	Moderate	30	Moderate	5-4	6-4	1-1	0-0
6	NS	E	High	30	High	6-4	8-3	3-2	0-0
7	CIE	E	Moderate	10	Moderate	4-3	7-5	4-4	0-0
8	CIE	E	Moderate	30	Moderate	3-3	5-3	2-2	0-0
9	CIE	E	Moderate	30	Moderate	5-4	7-5	9-8	4-4
10	KLICK	E	Moderate	10	Moderate	4-3	6-4	5-4	0-0
11	LI	S	Moderate	30	Moderate	9-7	2-1	6-5	8-8
12	LI	S	Moderate	20	Moderate	9-7	2-2	6-5	8-7
13	EK	E	Moderate	30	Moderate	7-7	6-4	6-5	0-0

*E* erythema, *S* scales/hyperkeratosis, *VAS* visual analogical scale

When introducing alitretinoin, 11 patients had been prescribed acitretin, which was consequently stopped. The median dosage of acitretin had been 0.4 mg/kg/day [range: 0.2-0.5] and the median duration of treatment was 84 months [range: 1-252]. The main reason for introducing alitretinoin was a desire to become pregnant for all ten female patients, of which two had a very severe disease (VAS scale: 9/10). The replacement of acitretin with alitretinoin was also because of insufficient treatment efficacy. All three males had been prescribed acitretin but wanted to change treatment because of its side effects (mucous dryness and myalgia) or insufficient efficacy. No changes to skin care were made during treatment with alitretinoin (Table 2). The starting dose of alitretinoin was 10 mg per day for all patients. For nine patients, this dose was increased after 1 month to 30 mg. For patient 12, dosage was only increased to 20 mg after 2 months because of headaches. The other three patients were maintained on 10 mg per day as the treatment was fully efficacious.

The reduction in scaling was not consistent between the five patients that had prominent scaling, with VAS either decreasing or increasing. All eight patients with prominent erythema had a reduction in VAS (median improvement of 2 points [range: 2-5]). Their scaling remained stable or slightly improved.

The assessment of other parameters (palmoplantar keratoderma or ectropion) did not reveal any significant improvement with treatment.

Alitretinoin global efficacy was considered low in two patients, moderate in nine and high in two patients. These latter two patients had lamellar ichthyosis caused by a *CYP4F22* mutation and Netherton syndrome, respectively (for these patients, the VAS erythema was reduced by 4 and 5 points, respectively).

Blood tests included assessment of liver-enzyme levels and a lipid profile (cholesterol, triglycerides) for all patients, and assessment of T4 and thyroid-stimulating hormone for six patients. The tests were performed for all patients at baseline, and then after 1 month and then at different time points (each month or every 3 months). *β*-HCG was assessed monthly in women of childbearing age. X-rays (spinal and pelvis) were performed on only three patients at baseline.

The reported side-effects were as follows: moderate to severe headaches (four patients, of which three were receiving 30 mg/day, required analgesics on demand; and one patient who presented spontaneous resolution of headaches after 8 weeks treatment with alitretinoin 10 mg/day, allowing drug dosage increase to 20 mg/day), oral mucous dryness (two patients receiving 10 or 30 mg/day), myalgia (one patient), benign intracranial hypertension presenting with headaches, blurred vision, and papilledema (an MRI of the brain was unremarkable), which completely resolved after withdrawal of alitretinoin (one patient). The benign intracranial hypertension was the only side effect that led to drug discontinuation. Alitretinoin was also discontinued in two patients that needed a decreased dose because of moderate headache and, consequently, they experienced inadequate treatment efficacy.

## Discussion

We report here on the largest series of patients with II and treated using alitretinoin. We show that alitretinoin was effective at reducing erythema and was otherwise well-tolerated.

There are some limitations to our study. One was related to the rarity of II, and thus the small number of included patients. The second limitation is related to the retrospective design of the study, with different dosages and treatment durations. We were also not able to compare the effect on alitretinoin to baseline data as most patients were receiving acitretin at the time when alitretinoin therapy was started.

In the literature, the effect and tolerance to alitretinoin has only been reported for six patients: a case series of four patients [11] and two case reports on KID (keratitis ichthyosis deafness) syndrome [9, 10].

The series by Gånemo et al., included four cases of II: one had epidermolytic ichthyosis (caused by a *KRT10* mutation) and three had lamellar ichthyosis (caused by a *TGM1* mutation). In contrast to our study, none of the cases had prominent erythema. All four patients had decreased scaling (no score provided) using high-dose alitretinoin (30-60 mg/day). However, this improvement was not better than previously observed with acitretin, except for the patient with epidermolytic ichthyosis. Dry lips were reported by all patients at the highest dose, and one experienced mild myalgia and another reported having headaches. Two patients had changed levels of thyroid hormone: one patient reverted to baseline values after alitretinoin was suspended, and the other showed signs of autoimmune hypothyroidism [11].

In the other studies, two patients with KID syndrome had reductions in hyperkeratosis and erythema and no significant side-effects when using a dosage of 20 or 30 mg/day of alitretinoin [9, 10].

In a series of 16 cases of epidermolytic ichthyosis, the efficacy of different oral retinoids, including alitretinoin, was reported. Only three patients had an acceptable response to treatment, but the effects of alitretinoin were not assessed separately and the dosage of alitretinoin was not mentioned [12].

**Fig. 1:**
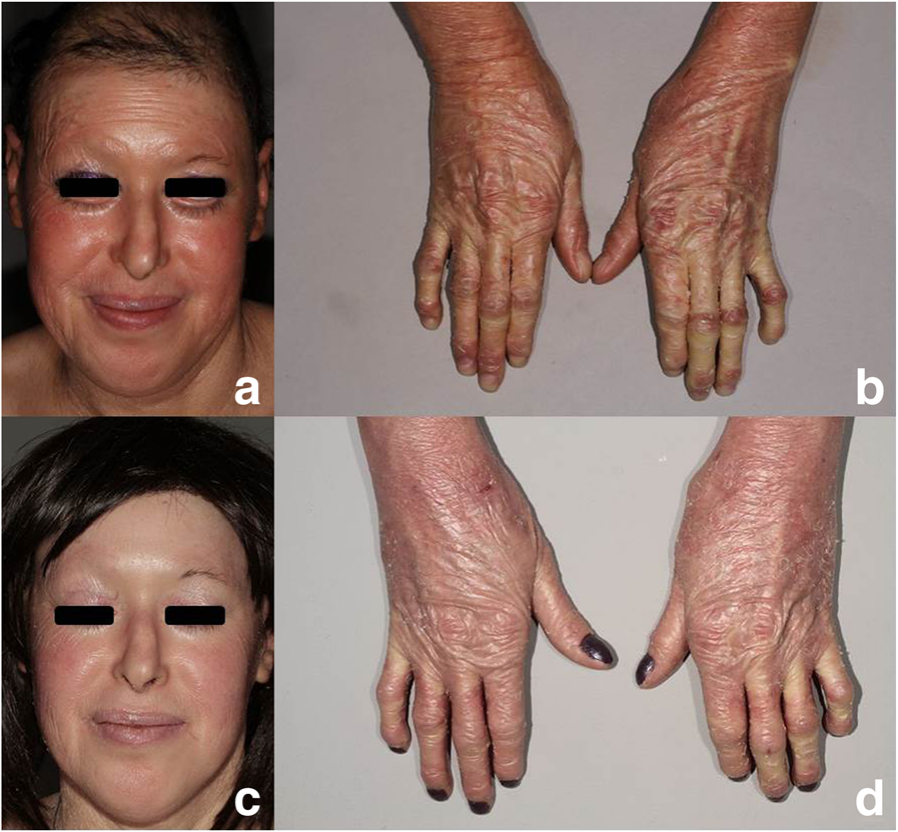
Patient 9 with congenital ichthyosiform erythroderma due to *ABCA12* mutation: comparison of the erythema on the face and dorsal aspects of the hands before alitretinoin (VAS 7) (**a**, **b**) and after 24 months on alitretinoin (dosage of 30 mg per day) (**c**, **d**)

**Fig. 2:**
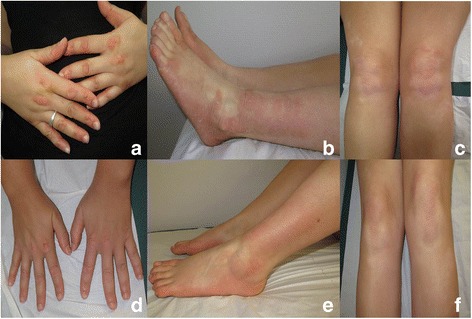
Patient 13 with erythrokeratoderma due to *GJB3* mutation: comparison of the erythema on the dorsal aspect of the hands, the external side of lower leg and ankle, the knees before alitretinoin (VAS 6) (**a**, **b**, **c**) and after 2 months on alitretinoin (dosage of 30 mg per day) (**c**, **d**, **e**)

All these data suggest that alitretinoin is not effective at reducing scaling or palmoplantar keratoderma, even at high doses. In contrast, alitretinoin seemed effective at reducing erythema (Figs. 1 and 2). The best VAS improvement (from 8 to 2) was experienced by the patient affected by NS, a clinical form of ichthyosis which is particularly characterized by erythroderma. This efficacy on erythema seems to be fast since it was seen after only 1 or 2 months for patients 2,12,13.

The fact that alitretinoin improves erythema enhances the hypothesis of an anti-inflammatory effect. This is in accordance with efficacy of alitretinoin to reduce erythema in patients that have chronic hand eczema [7] or KID syndrome [9, 10].

Alitretinoin thus seems to be suitable to treat II associated with prominent erythema. The dosage of 10 mg per day may be sufficient for some patients, whereas others may require higher doses (up to 30 mg). There are no published data on the effect of alitretinoin on ectropion. In our series, only three patients had ectropion and remained stable under alitretinoin.

It was intriguing to note that two sisters with similar phenotypes (patients 3 and 4) had different skin responses (improvement or worsening) using a similar dosage of alitretinoin, suggesting inter-individual variability in drug response. Some authors suggest that the response to retinoids should also be influenced by the causal gene: patients with epidermolytic ichthyosis caused by a *KRT10* mutation responding better than those with a *KRT1* mutation [11, 13].

The side-effects reported with alitretinoin do not seem to differ from those with acitretin, except for thyroid dysfunction (not reported in our series) and headache (more frequent with alitretinoin). These side-effects occasionally led to discontinuation of treatment. The most serious side-effect was seen in a patient with Netherton syndrome where treatment had to be interrupted despite excellent improvement in skin features.

Due to a paucity of data in the literature, the exact incidence of benign intracranial hypertension (pseudotumor cerebri) is unknown. Safety analysis of the pivotal studies and postmarketing adverse events since alitretinoin approval, only revealed eight cases of benign intracranial hypertension [14]. This side-effect may be observed in patients taking other drugs (such as tetracycline), concomitantly [15].

## Conclusion

Alitretinoin may be suitable for those with II and prominent erythema, especially for women of childbearing age. A dosage of 10 mg per day may be sufficient in some cases. The risk/benefit balance of alitretinoin seems favorable, but monitoring is necessary, as is recommended for other similar diseases or for acitretin therapy [7, 9, 11]. This prescription remains off-label and larger prospective controlled studies are needed to confirm these results.

## Abbreviations

AC: Acitretin
AL: Alitretinoin
CIE: Congenital ichthyosiform erythroderma
E: Erythema
EK: Erythrokeratoderma
IE: Insufficient efficacy
II: Inherited ichthyosis
KLICK: Keratosis linearis ichthyosis congenital keratoderma
LI: Lamellar ichthyosis
M: Months
NS: Netherton syndrome
RD: Reduced dosage
S: Scales/hyperkeratosis (Table II)
S: Stopped treatment (Table I)
SE: Side-effects
VAS: Visual analog scales
Y: years

## References

[1] Traupe H, Fischer J, Oji V (2014). Non-syndromic types of ichthyoses - an update. J Dtsch Dermatol Ges.

[2] Oji V, Tadini G, Akiyama M, Blanchet Bardon C, Bodemer C, Bourrat E (2010). Revised nomenclature and classification of inherited ichthyoses: results of the first ichthyosis consensus conference in Sorèze 2009. J Am Acad Dermatol.

[3] Mazereeuw-Hautier J, Dreyfus I, Barbarot S, Serrentino L, Bourdon-Lanoy E, Ezzedine K (2012). Factors influencing quality of life in patients with inherited ichthyosis: a qualitative study using focus groups. Br J Dermatol.

[4] Dreyfus I, Bourrat E, Maruani A, Bessis D, Chiavérini C, Vabres P (2014). Factors associated with impaired quality of life in adult patients suffering from ichthyosis. Acta Derm Venereol.

[5] Ormerod AD, Campalani E, Goodfield MJ (2010). BAD clinical standards unit. British Association of Dermatologists guidelines on the efficacy and use of acitretin in dermatology. Br J Dermatol.

[6] Vahlquist A, Lööf L, Nordlinder H, Rollman O, Vahlquist C (1985). Differential hepatotoxicity of two oral retinoids (etretinate and isotretinoin) in a patient with palmoplantar psoriasis. Acta Derm Venereol.

[7] Blair HA, Scott LJ (2016). Alitretinoin: a review in severe chronic hand eczema. Drugs.

[8] Ruzicka T, Lynde CW, Jemec GB, Diepgen T, Berth-Jones J, Coenraads PJ (2008). Efficacy and safety of oral alitretinoin (9-cis retinoic acid) in patients with severe chronic hand eczema refractory to topical corticosteroids: results of a randomized, double-blind, placebo-controlled, multicentre trial. Br J Dermatol.

[9] Prasad SC, Bygum A (2013). Successful treatment with alitretinoin of dissecting cellulitis of the scalp in keratitis-ichthyosis-deafness syndrome. Acta Derm Venereol.

[10] Werchau S, Toberer F, Enk A, Hembold P (2011). Keratitis-ichthyosis-deafness syndrome: response to alitretinoin and review of literature. Arch Dermatol.

[11] Gånemo A, Sommerlund M, Vahlquist A (2012). Oral alitretinoin in congenital ichthyosis: a pilot study shows variable effects and a risk of central hypothyroidism. Acta Derm Venereol.

[12] Bygum A, Virtanen M, Brandrup F, Gånemo A, Sommerlund M, Strauss G (2013). Generalized and naevoid epidermolytic ichthyosis in Denmark: clinical and mutational findings. Acta Derm Venereol.

[13] Virtanen M, Gedde-Dahl T, Mörk NJ, Leigh I, Bowden PE, Vahlquist A (2001). Phenotypic/genotypic correlations in patients with epidermolytic hyperkeratosis and the effects of retinoid therapy on keratin expression. Acta Derm Venereol.

[14] Morris M, Schifano L, Fong R, Graff O (2016). Safety of alitretinoin for severe refractory chronic hand eczema: clinical studies and postmarketing surveillance. J Dermatolog Treat.

[15] Chroni E, Monastirli A, Tsambaos D (2010). Neuromuscular adverse effects associated with systemic retinoid dermatotherapy: monitoring and treatment algorithm for clinicians. Drug Saf.

